# Circular Rep-Encoding Single-Stranded DNA Sequences in Milk from Water Buffaloes (*Bubalus arnee f. bubalis*)

**DOI:** 10.3390/v13061088

**Published:** 2021-06-07

**Authors:** Marie-T. König, Robert Fux, Ellen Link, Gerd Sutter, Erwin Märtlbauer, Andrea Didier

**Affiliations:** 1 Department of Veterinary Sciences, Institute of Food Safety, Faculty of Veterinary Medicine, Ludwig-Maximilians-Universität München, Schönleutnerstraße 8, 85764 Oberschleißheim, Germany; marie-therese.koenig@mh.vetmed.uni-muenchen.de (M.-T.K.); milchhygiene@mh.vetmed.uni-muenchen.de (E.M.); 2 Department of Veterinary Sciences, Chair of Virology, Faculty of Veterinary Medicine, Ludwig-Maximilians-Universität München, Veterinärstraße 13, 80539 München, Germany; robert.fux@micro.vetmed.uni-muenchen.de (R.F.); ellen.link@micro.vetmed.uni-muenchen.de (E.L.); gerd.sutter@lmu.de (G.S.)

**Keywords:** BMMF, circular ssDNA, colon/breast cancer, replication-associated protein (Rep), rolling circle amplification, rolling circle replication, water buffalo milk

## Abstract

Isolation and characterization of circular replicase-encoding single-stranded (ss) DNA from animal, plant and environmental samples are rapidly evolving in virology. We detected 21 circular DNA elements, including one genomoviral sequence, in individual milk samples from domesticated Asian water buffaloes (*Bubalus arnee f. bubalis*). Most of the obtained genomes are related to Sphinx 1.76 and Sphinx 2.36 sequences and share a high degree of similarity to recently published circular DNAs—named BMMF (bovine meat and milk factors)—that have been isolated from commercial milk, as well as from bovine serum. Characteristic features such as *rep* genes, tandem repeats and inverted repeats were detected. These BMMF have recently been found to be present in taurine-type dairy cattle breeds descending from the aurochs (*Bos primigenius*). Importantly, the occurrence of BMMF has been linked to the higher incidence of colorectal and breast cancer in North America and Western Europe compared with Asia. This is the first report of circular ssDNA detected in milk from the domesticated form of the wild Asian water buffalo (*B. arnee*) belonging to the subfamily *Bovinae*. This novelty should be taken into account in view of the above-mentioned cancer hypothesis.

## 1. Introduction

Single-stranded (ss) DNA viruses can be detected throughout all domains of life and are currently classified into thirteen families according to the International Committee on Taxonomy of Viruses (ICTV) [1]. Reports on unclassified virus-like ssDNA have drastically increased in recent years [2,3]. This augmentation is mainly caused by the application of high-throughput metagenomics such as next-generation sequencing (NGS) [4]. Nevertheless, classical approaches comprising rolling circle amplification (RCA) [5] followed by PCR and Sanger sequencing still have their importance for sequence detection and characterization. Recently, these sequences have been assigned to the virus phylum *Cressdnaviricota*, where CRESS stands for “circular Rep-encoding single-stranded”, to describe functional features of these isolates, and viricota is the suffix for phylum taxa [6]. Besides their circular ssDNA composition, they depend on a replicase (Rep) for rolling circle replication (RCR) of their genomes. Encoded Reps exhibit a nicking/joining activity at the origin of replication (ori) of the ssDNAs. In addition to this endonuclease domain at the N-terminus, in some cases Reps include a superfamily 3 (SF3) helicase domain at their C-terminus, which helps to unwind the double-stranded DNA replicative intermediate [7,8]. In other cases, helicases encoded by the host are recruited during RCR. Sequence analysis of Reps on the genetic as well as on the protein level provides information about the evolutionary history and the phylogenetic relatedness of different CRESS isolates [9]. The presence of a replicase is not restricted to CRESS DNA viruses, with sometimes blurred borders between CRESS viruses and plasmid-/phage-derived sequences. A prominent example of this overlap is the co-purification of Sphinx 1.76 and Sphinx 2.36 from transmissible spongiform encephalopathy (TSE) particles [10]. The first isolate shows significant sequence homology to a plasmid isolated from *Acinetobacter baumannii*, while the latter one shows similarities to an *Acinetobacter* sp. phage [11]. The resemblance between these sequences and their common mechanism of replication led to the hypothesis that ssDNA viruses developed from bacterial plasmids via gene transfer with RNA viruses [12,13]. Recently, Sphinx 1.76-related and Sphinx 2.36-related circular DNAs have also been isolated from cattle milk, cattle serum and human brain affected by multiple sclerosis [14,15,16]. With the ongoing identification of these types of ssDNA from bovine samples, the term BMMF (bovine meat and milk factors) has been introduced, and these factors have been suggested to represent a specific class of infectious agents [17]. According to their sequence homologies to known organisms, the factors are divided into four groups. The first two groups consist of isolates resembling the sequences of Sphinx 1.76 (group 1) or Sphinx 2.36 (group 2). Group 3 contains isolates that have been characterized as novel gemycircularviruses, being one of nine genera within the family *Genomoviridae*. A single isolate with similarity to a *Psychrobacter* species plasmid forms group 4. The terms HCBI (healthy cattle blood isolate) and CMI (cow milk isolate) are utilized to refer to the origin of each isolate [11]. A common attribute on the DNA level of all group 1 isolates is the presence of an iteron-like tandem repeat, a neighbouring inverted repeat, and at least one large open reading frame (ORF)-encoding Rep. Some of the isolates encode a putative second ORF resembling a mobilization element [18]. In contrast to other animal-derived CRESS viruses, these circular DNAs lack the genetic information for a capsid protein.

From a disease-related point of view, BMMF and Sphinx-like sequences have entered the spotlight due to a potential connection with colorectal and breast cancer as well as neurodegenerative illnesses such as multiple sclerosis [19]. BMMF DNA and Rep proteins have been found in tissue of colon cancer patients and are proposed to cause chronic inflammation [20], thus linking the cancer hypothesis to the consumption of milk and meat from taurine cattle. These are descendants from the European aurochs and are potential carriers of BMMF [21]. In North America and Western Europe, cattle breeds are largely taurine in origin (*Bos taurus taurus*). In contrast, the Asian cattle population largely consists of cattle breeds derived from the indicine lineage (*Bos taurus indicus*) [22,23], yaks *(Bos mutus)* [24] and water buffaloes (*Bubalus arnee)* [25], which are not supposed to be carriers of BMMF [26]. Especially on the Indian subcontinent and in Mongolia, the above-mentioned cancer incidence is lower than in Western countries [21,27]. However, studies on samples of “Asian-type” cattle investigating the presence of circular replication competent ssDNA sequences are missing in the literature. For this reason, we initiated an in-depth analysis of individual milk samples obtained from Asian water buffaloes (*B. arnee f. bubalis*).

## 2. Methods

### 2.1. Sample Collection

In total, 30 individual milk samples (50 mL) were collected from two different buffalo flocks in Germany, one kept in Lower Saxony (A) and one in Baden-Württemberg (B). A total of 23 samples were obtained from herd A and 7 from herd B. Immediately after collection, the milk was cooled to 4 °C and cold-shipped to the Institute of Food Safety within 24 h where it was immediately processed. Samples from flock A were numbered 8 through 30 and from flock B 42 through 48. In the case of obtaining full-length circular DNA sequences, sample numbers were completed with the term BAMI, which refers to “Bubalus Arnee Milk Isolate” and a number indicating the sequence length. 

### 2.2. DNA Extraction and Rolling Circle Amplification (RCA) of Circular ssDNA

The QIAamp DNA Mini Kit (Qiagen, Hilden, Germany) was applied to isolate total DNA from 200 μL of whole milk according to the manufacturer’s instructions. Approx. 40 ng of purified DNA was subjected to RCA using the TempliPhi Amplification Kit (Cytiva, Marlborough, MA, USA) with random primers according to the instruction manual of the supplier. Isothermal amplification was carried out at 30 °C for 18 h.

### 2.3. PCR Screening

RCA products were initially screened for the presence of *rep*-sequences with a set of 10 primer pairs designed via the *rep* genes of Sphinx 1.76 (GenBank Acc. No. HQ444404.1), Sphinx 2.36 (GenBank Acc. No. HQ444405.1) and related sequences obtained by Blast search. A similar approach was chosen with primers for *Genomoviridae*. The total volume of all PCR reactions was 50 μL applied on a TProfessional Gradient 96 Thermocycler (Biometra, Jena, Germany). ThermoPrime Plus Polymerase (ThermoScientific, Waltham, MA, USA) was used at 1.25 U/reaction. The dNTPs (ThermoScientific, Waltham, MA, USA) were added to a final concentration of 200 μM each. Detailed information on the primer sequences as well as the individual amplification programmes is summarized in Tables S1 and S2. After agarose-gel electrophoresis, bands were purified with the HighYield PCR Purification/Gel Extraction Kit (SLG, Südlaborbedarf, Gauting, Germany). Sequencing was performed by MWG Eurofins (Ebersberg, Germany). The sequences obtained from the PCR screening were aligned, and primer pairs matching homologous regions of the alignments were designed for inverse PCR in order to obtain full-length sequences of the circular DNA elements (for detailed information see Table S3). Additionally, abutting primers for amplification of BMMF2 sequences published by de Villiers et al. [18] and for BMMF1 sequences published by Whitley et al. [16] were applied.

### 2.4. Cloning of Inverse PCR Products

Amplification products obtained from inverse PCR were cloned into pCR2.1 TopoTA vector (Invitrogen, Carlsbad, CA, USA). Briefly, the gel-purified PCR product was mixed with the vector at a molar ratio of approx. 3:1 in a total volume of 6 μL and allowed to rest for 30 min at room temperature for ligation. Afterwards, chemically competent *Escherichia coli* DH5 alpha were transformed with the ligation reaction for 30 min on ice. After a heat shock at 42 °C for 1 min, 1 mL of LB medium was added to the reaction and cells were allowed to recover for 1 h at 37 °C with gentle agitation. Next, *E. coli* were centrifuged at 6100× *g* for 5 min. The pellet was suspended in 200 μL LB medium and plated on LB agar with ampicillin (100 μg/mL). After overnight incubation in LB medium with ampicillin, five clones from each plate were subjected to MiniPrep Plasmid isolation via the GenElute Plasmid Miniprep Kit (Sigma Aldrich, Taufkirchen, Germany). A control digest of 500 ng plasmid DNA with EcoRI (New England BioLabs, Ipswich, MA, USA) was performed, and insert-bearing clones were sequenced via the M13for and M13rev primer binding sites present in the vector.

### 2.5. Data Analysis

Sequences obtained from MWG Eurofins were subjected to BlastSearch against the nucleotide database at NCBI [28]. For multiple sequence alignments and cross-comparison, the DNASTAR Software (DNASTAR Inc., Madison, WI, USA, Version 15.0.0) was used [29]. Phylogenetic trees were calculated in MEGA software (version 5.03) by using the maximum likelihood method, and bootstrap values were computed with 500 replicates [30]. Furthermore, all sequences were checked at Emboss explorer for tandem repeats [31] and inverted repeats [32] upstream the *rep* gene sequence. Maximum size of tandem repeats was restricted to 30 nucleotides (nt), and the minimum size of inverted repeats was set to 4 nt. In silico detection of potential open reading frames was performed with the ORFfinder software at NCBI [33]. ATG or TTG as an alternative start codon was allowed. The minimum ORF size was restricted to 75 amino acids (aa). For subsequent comparative and functional analysis, only ORFs ≥ 95 aa were included. All full-length genomes and their annotations detected in this study were deposited in the GenBank database (Accession numbers MW828657–MW828677).

## 3. Results

### 3.1. PCR Screening and Detection of Circular Single-Stranded DNA Elements

By using RCA followed by several screening PCRs, 83% (19/23) of the individual milk samples from flock A and 100% (7/7) from flock B reacted positive in at least one PCR applied for BMMF and CRESS DNA detection. Inverse PCRs employing novel primers from this study resulted in retrieval of four circular DNA elements, all from flock A (8BAMI.2351, 11BAMI.2199, 12BAMI.2349, 21BAMI.2076). Application of abutting primers published by Whitley et al. [16] and de Villiers et al. [18] yielded 17 additional circular DNAs from samples of both flocks. Interestingly, five of the positive milk samples (8BAMI, 11BAMI, 12BAMI, 21BAMI, 22BAMI) contained two different circular DNA elements. Altogether, 21 unique complete circular ssDNA genomes were detected in the present study, one of them later assigned as gemycircularvirus.

### 3.2. Characterization of the Sequences on the DNA Level

The overall sequence length of the circular DNA elements ranged from 1972 to 2522 nt. After subjecting all DNAs to BlastSearch, the best two matches were summarized, as shown in Table S4. Nine of the 21 detected circular DNAs are nearly identical to the BMMF1 isolates HCBI6.252 and CMI1.252, showing a query coverage of 100% and more than 99% identity. Seven sequences are similar to C1MI GenBank entries with a query coverage of 71–98% and 77–97% identity, respectively. Four of the DNA elements revealed similarities to C2MI isolates, belonging to the BMMF2 group. One sequence from the present study was assigned to a gemycircularvirus detected in a giant panda with 92.94% identity in BlastSearch but 48% query coverage only and therefore belonged to the BMMF group 3. None of the herein detected isolates belongs to BMMF group 4.

Homologies among sequences from the present study were calculated by additional multiple sequence alignment. Sequences 8BAMI.2351, 12BAMI.2349, 21BAMI.2076 and 44BAMI.2371 are the most remote ssDNA elements showing a maximum of 41% identity to the other sequences, but 59–99% identity among each other. Some of the other circular DNAs (8BAMI.2522, 9BAMI.2522, 15BAMI.2522, 17BAMI.2522, 22BAMI.2521, 22BAMI.2522, 45BAMI.2521, 46BAMI.2522) share ≥ 99% identity despite originating from different flocks. These isolates differ in 6 to 14 nt positions only. A similar observation was made for isolates 11BAMI.2092, 12BAMI.2092, 13BAMI.2092, 21BAMI.2091 and 24BAMI.2092, which possess ≥ 99% identity as well.

**Figure 1:**
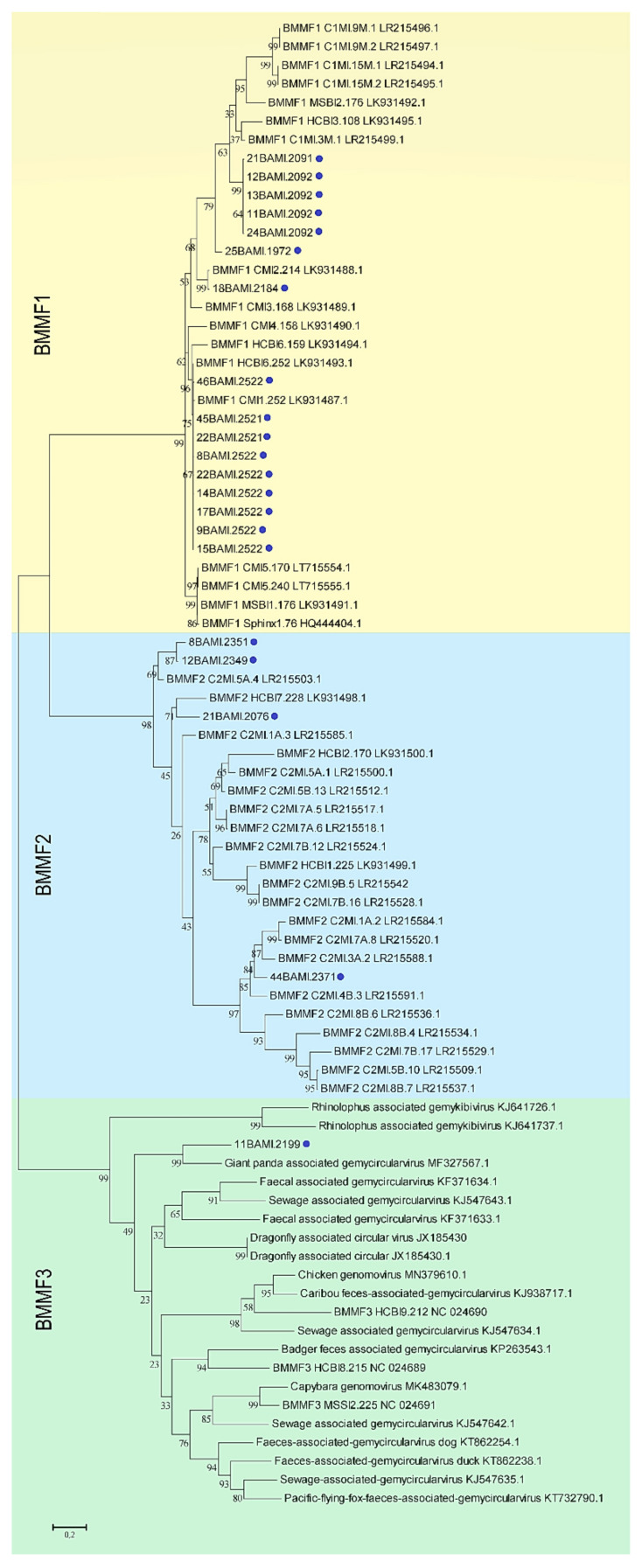
Results of the molecular phylogenetic analysis including novel full-length nucleotide sequences and representatives of the BMMF groups 1 to 3. The latter also includes members of the family *Genomoviridae*. The evolutionary history was inferred by using the maximum likelihood method and a JTT matrix-based model [34]. The bootstrap consensus tree was calculated from 500 replicates. Bootstrap values at the branch points indicate the percentage of replicate trees in which respective isolates clustered [35]. Branch support values lower than 70% were not included. Blue dots mark all sequences found in this study. The scale bar represents the number of substitutions per site. All taxa are indicated by name followed by the corresponding GenBank accession number.

Next, a phylogenetic distance tree was generated including Sphinx 1.76, Sphinx 2.36, representatives from the BMMF1 and 2 groups and from the family *Genomoviridae* as references (Figure 1). This approach allowed the affiliation of the novel sequences to the BMMF1 and 2 groups. Within the BMMF1 group, the isolates 11BAMI.2092, 12BAMI.2092, 13BAMI.2092, 21BAMI.2091 and 24BAMI.2092 form a cluster distinct from any of the sequences published by the group of zur Hausen so far. The Gemycircularvirus-like element detected in sample 11 is the only representative of the BMMF3 group according to the nomenclature of zur Hausen et al. [11].

Further analysis of the 21 circular sequences for typical replication-relevant ssDNA elements included: (i) search for tandem repeats (TR) [31], (ii) search for inverted repeats (IR)/stem loops as a potential origin of replication [32], (iii) search for replicase genes and (iv) search for additional putative ORFs [33].

**Figure 2:**
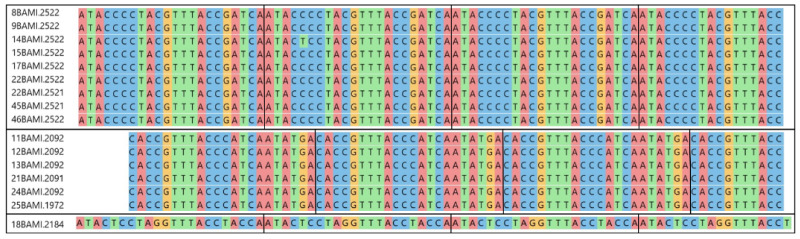
Clustal alignment of the three different TR motifs identified. Each of the upper two boxes comprises isolates with identical TR sequences, except for one nucleotide substitution in 14BAMI.2522. The lower box shows one further TR represented by a single isolate only. Vertical black lines indicate the end of a single repeat. The TR size is 22 nt, and the copy number ranges from 3.5 to 3.9.

Except for the circular DNA sequences 8BAMI.2351, 12BAMI.2349, 21BAMI.2076 and 44.BAMI.2371, all isolates exhibited typical tandem repeats that can also be found in related BMMF [11]. The DNA elements lacking a tandem repeat belonged either to the BMMF2 or BMMF3 group. Detailed information on the tandem repeat features is illustrated in Figure 2.

Furthermore, the circular DNAs were analysed for the presence of inverted repeats in proximity to the tandem repeat region, thus potentially able to form a double strand and to function as a replicase docking site. All BMMF1 sequences with TR motifs contain the same palindromic sequence TAAATGCTTTTA (the first and the last 4 nt build the inverted repeat region separated by a 4 nt gap), which is located 49 to 56 nt upstream of the TR region (Table 1) and has also been described by zur Hausen et al. [11].

### 3.3. In Silico Characterization of BMMF-like DNA Elements on the Protein Level

**Figure 3:**
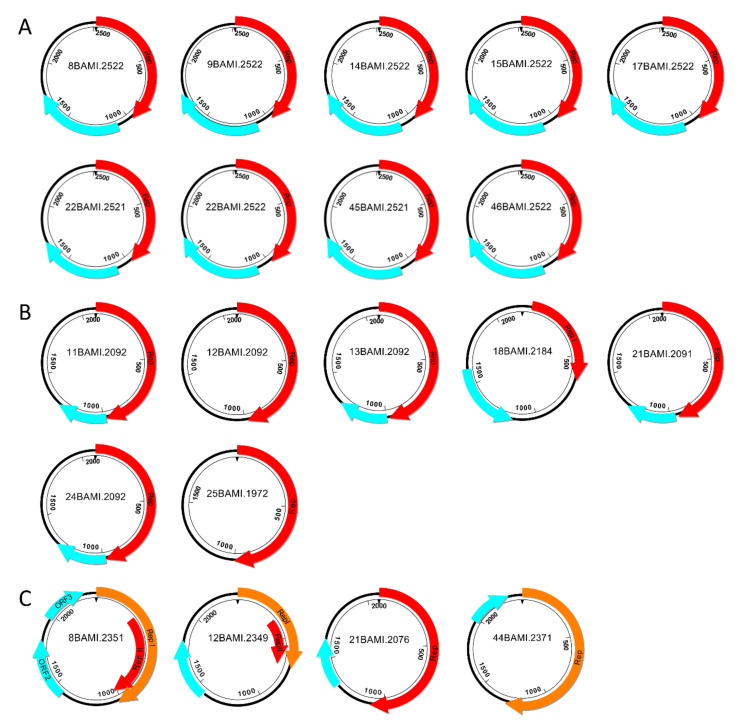
Schematic illustration of BMMF-like DNA elements recovered from buffalo milk. Red arrows indicate *rep* genes starting with ATG; orange arrows show *rep* genes starting with TTG. The light blue arrows represent further potential ORFs. According to the results of the in silico analysis, circular DNA elements were assigned to three groups (**A**–**C**). Group (**A**) contains nine related isolates, which could be assigned to the GenBank entries CMI1.252 and HCBI6.252; group (**B**) comprises seven elements, which exhibit the greatest homologies to other BMMF1 isolates; group (**C**) includes four circular DNAs belonging to the BMMF2 group.

In addition to the analyses on the nucleotide level, we performed an in silico characterization in order to detect open reading frames (ORFs) [33] and the deduced proteins including a subsequent BlastSearch. Basic features of all circular DNA elements detected in this study are depicted in Figure 3. The only gemycircularvirus found herein is described in Section 3.4.

Each circular DNA element possesses an ORF coding for Rep. Depending on the settings in the ORFfinder tool (“ATG only” versus “ATG and alternative initiation codons”), results slightly differ. Therefore, two isolates with overlapping or nested *rep*-ORFs were found, starting with either ATG or TTG. In this case, respective protein sequences were named as follows: 8BAMI.2351.RepI and 8BAMI.2351.RepII. Results of BlastSearch analysis are included in Table S5.

**Figure 4:**
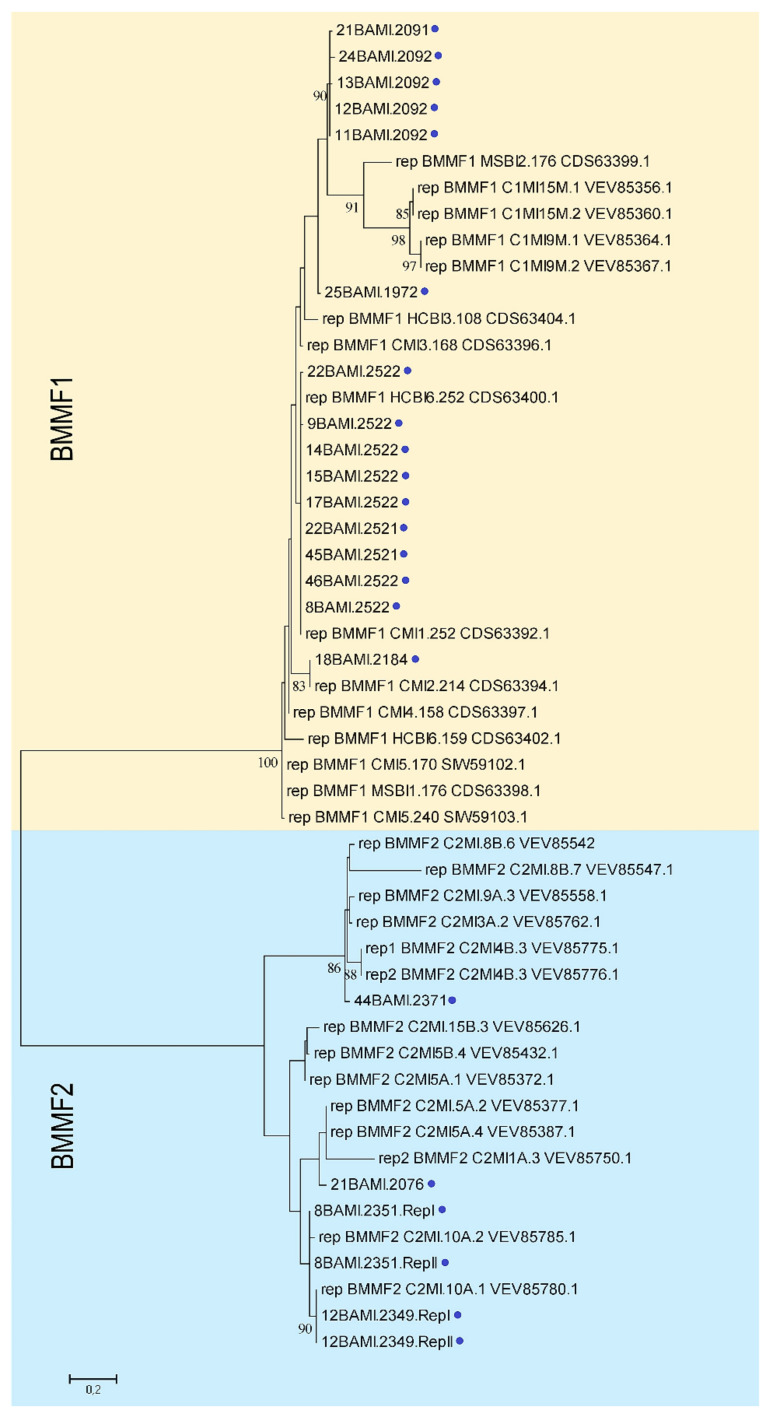
Maximum likelihood phylogenetic tree of Rep amino acid sequences and representative Reps from BMMF1 and 2 isolates calculated as described in Figure 1 [36]. The bootstrap consensus tree is based on 500 replicates. Bootstrap values at the branch points indicate the percentage of replicate trees in which respective isolates clustered [35]. Branch support values lower than 70% were not included. Blue dots mark all sequences found in this study. The scale bar represents the number of substitutions per site. All taxa are indicated by name followed by the corresponding GenBank accession number.

The majority (13) of the 22 putative Reps found in this study showed the best Blast hits for BMMF-related replicases. The remaining nine Reps scored closer to *Acinetobacter*-related replicases or those from even more distant organisms. Remarkably, the *rep* translation products of 12BAMI.2349 and 18BAMI.2184 are shorter (up to 150 aa) than their best matches after BlastSearch (Table S5). Phylogenetic analysis of the Rep proteins revealed that they belong to the established BMMF groups (Figure 4). Again, the isolates 11BAMI.2092, 12BAMI.2092, 13BAMI.2092, 21BAMI.2091 and 24BAMI.2092 build their own cluster.

All DNA elements were analysed for the potential occurrence of further proteins. Although the software tool detected up to 22 putative ORFs per DNA sequence, the length of the potential proteins was restricted to a minimum of 95 aa to be included in the Results section of the manuscript. For additional putative proteins, the same approach as for the Reps was carried out. Thirteen putative proteins found in the novel sequences were identical or nearly identical to uncharacterized proteins from BMMF1 or BMMF2 isolates. Detailed results of the BlastSearch are summarized in Table S6. 12BAMI.2092 and 25BAMI.1972 do not code for an additional, hypothetical protein. A phylogenetic tree based on the putative protein sequences is given in Figure S1.

### 3.4. In Silico Characterization of a Novel Gemycircularvirus on the Protein Level

**Figure 5:**
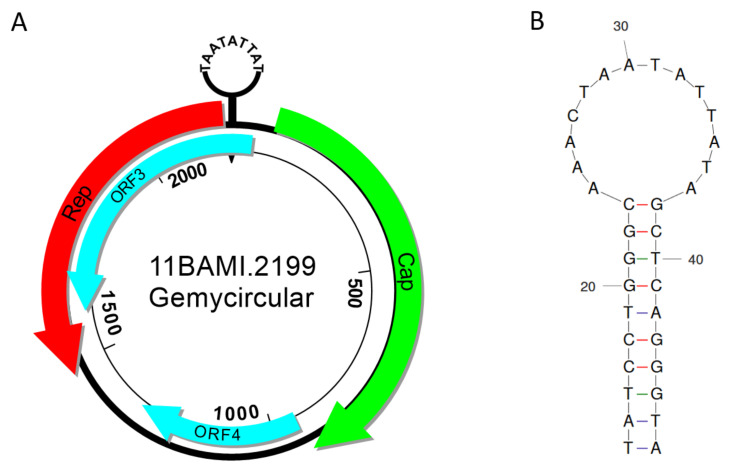
(**A**) Schematic map of the gemycircularvirus 11BAMI.2199. The in silico generated genes encoding Rep and Cap are shown as red and green arrows, respectively. Further putative ORFs are shown as light blue arrows. The nonanucleotide motif (TAATATTAT) within the potential stem-loop structure at position 1 is depicted at the top. (**B**) Enlarged view of the stem-loop structure between *rep* and *cap* as a potential origin of replication as calculated by the ProbKnot server at the University of Rochester. The nucleic acid type was set to DNA (http://rna.urmc.rochester.edu/RNAstructureWeb/Servers/ProbKnot/ProbKnot.html; accessed on 17 March 2021).

The only CRESS DNA virus isolated herein was recovered from a buffalo milk sample also containing one more circular DNA element belonging to the BMMF1 group. After in silico analyses, this new CRESS DNA virus was named 11BAMI.2199 (GenBank accession number MW828677). Typical characteristics of a gemycircularvirus could be identified. Besides a stem-loop structure at the origin of replication, it includes a *capsid* (*cap*) gene on the sense strand and a *rep* gene on the antisense strand. The putative stem-loop structure in the intergenic region between *rep* and *cap* features a nonanucleotide motif (TAATATTAT) that is present at the loop-tip and therefore a further characteristic of *Genomoviridae* [37,38,39] (Figure 5). Additionally, the isolate comprises two putative proteins, one in sense, the other one in antisense direction.

BlastSearches of the proteins encoded by this virus revealed 60–93% identity to giant panda-associated and plant-associated proteins, inter alia (GenBank entries: QCX29436.1, ASH99166.1, ASH99165.1).

**Figure 6:**
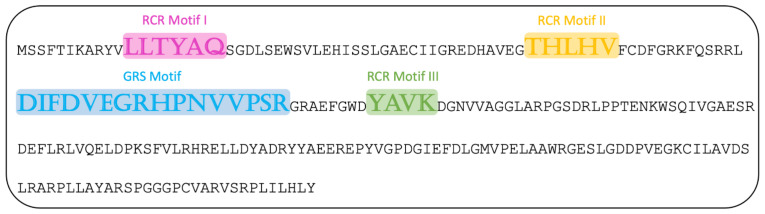
Conserved motifs in the Rep of the *Gemycircularvirus* found in this study in accordance with data by Varsani et al. [38].

The typical replication mechanism for all CRESS DNA viruses is the rolling circle replication initiated by the Rep. 11BAMI.2199 encodes a Rep that contains well-conserved RCR motifs (Figure 6). Motif I is supposed to have a sequence recognition function [38]. Motif II is characteristic of HUH endonucleases [40]. Motif III is possibly involved in dsDNA nicking [38,41]. The GRS motif has initially been described in geminiviruses by Nash et al. [42].

## 4. Discussion

BMMF initially described by zur Hausen et al. [11,17] are suspected to correlate with colon and breast cancer incidences as well as neurodegenerative diseases in the “Western” world. BMMF DNA and Rep proteins have been detected in colorectal cancer peritumour and tumour tissues of human patients by immunohistochemistry and Western blot [20]. Thereby and by the means of transfection assays, transcriptional activity of BMMF can be assumed [43]. Lower cancer incidences in countries traditionally breeding cattle evolved from other bovine lineages such as water buffaloes, yaks and zebus suggest that circular ssDNA elements in milk from taurine cattle might be one of several food-borne risk factors that contribute to the aforementioned diseases.

The present study was initiated to screen milk samples from non-taurine cattle for the presence of BMMF and CRESS viruses. Although screening PCR results suggested that 86% (26/30) of the water buffalo milk samples were positive, circular full-length sequences could only be recovered from 53% (16/30). This discrepancy might be caused by the formation of linear by-products, which occur to some extent during RCA. Thus, a PCR screening with primers, amplifying solely subgenomic fragments, might not be a suitable marker for the potential presence of full-length genomes. Of the 21 circular ssDNA elements detected and characterized herein, 20 belong to the BMMF groups 1 and 2. Inverse PCR additionally revealed one sequence belonging to the genus *Gemycircularvirus* (BMMF3). The further in silico analysis of genomes described herein revealed that two sequences (12BAMI.2349 and 18BAMI.2184) contained Reps substantially reduced in length (Table S5). Their replication capacity might thus be questionable due to a potentially dysfunctional Rep. On the other hand, five of the 21 positive samples harbour two ssDNA elements. Therefore, one must also consider that a functional Rep from one element might be able to complement a dysfunctional Rep from a second sequence, thus enabling RCR of both. However, to answer this question, the functional activity and replication behaviour of these DNA elements will have to be tested in transfection assays, as described by Eilebrecht et al. [43].

From a nutritional point of view, water buffalo milk is of great importance in some Asian countries. Although the multipurpose domestic water buffalo is widely distributed all over the world [44], the largest number of animals kept for dairy production is recorded in India, contributing to approx. 68% of the world’s buffalo milk production [45]. Buffalo milk production in India exceeded 86 million tonnes in 2017–2018; at 49%, this accounts for the largest part of total Indian milk production, compared with cow’s milk at only 47%. The per capita availability of milk adds up to 337 g per day, and the domestic demand for milk and milk products is steadily increasing [46]. From the centre of domestication in the Indian subcontinent, domestic water buffaloes spread westward to Egypt, the Balkans and Italy around the 7th century [25]. Nowadays, significant flocks in Europe are mainly kept in Italy, Romania and Turkey, but they represent only a minor proportion of total livestock [47,48].

Apart from one study in Switzerland that found one *gemycircularvirus* in the plasma of water buffaloes [49], reports on “Asian-type” cattle harbouring CRESS viruses or even BMMF are scarce. Either the cattle under study descend from the taurine lineage [50,51] or authors do not provide substantial information on the breeds [52,53,54]. None of the previous studies address the occurrence of BMMF in “Asian-type” cattle such as water buffaloes. Hence, this is the first profound survey investigating the appearance of circular ssDNA, particularly that of BMMF.

Although data from the present study underpin that BMMF are not restricted to taurine cattle, one has to keep in mind that water buffaloes from “Western” flocks might have acquired these DNA elements due to their coexistence with taurine cattle by yet unknown routes of transmission. As water buffaloes in Germany typically have access to pasture, the transmission from neighbouring, taurine cattle via vectors such as black flies, for example, cannot be excluded. To clarify this issue, future studies on samples from water buffaloes domiciled in Asia are needed.

## Figures and Tables

**Table 1:** Inverted repeat motif as a potential origin of replication. Sequences were detected by EMBOSS inverted repeat finder and additionally compared with the sequences described by zur Hausen et al. [11].

Isolate	Size	Inverted Repeat	nt between IR and TR	nt between IR and *rep*
8BAMI.2522	12	TAAATGCTTTTA	50	193
9BAMI.2522	12		50	193
11BAMI.2092	12		56	160
12BAMI.2092	12		56	160
13BAMI.2092	12		56	160
14BAMI.2522	12		50	193
15BAMI.2522	12		50	193
17BAMI.2522	12		50	193
18BAMI.2184	12		49	133
21BAMI.2091	12		56	192
22BAMI.2522	12		50	193
22BAMI.2521	12		50	193
24BAMI.2092	12		56	160
25BAMI.1972	12		56	160
45BAMI.2521	12		50	193
46BAMI.2522	12		50	193

## Supplementary Materials

The following are available online at https://www.mdpi.com/article/10.3390/v13061088/s1, Figure S1: Maximum likelihood phylogenetic tree of novel hypothetical protein sequences and uncharacterized BMMF1 and 2 proteins; Table S1: Information on the primer sets applied in the PCR screening for the amplification of Sphinx-like sequences. Table S2: Information on the primer sets applied in the PCR screening for the amplification of *Genomoviridae* family members. Table S3: Information on the primer sets applied in the inverse PCR for amplification of full-length DNA. Table S4: Summary of the BLASTn results for the individual isolates. Table S5: Summary of the BLASTp results of the in silico generated Reps. Table S6: Summary of the BLASTp results of the in silico generated additional proteins.
