# Supplementary material for: Circular Rep-Encoding Single-Stranded DNA Sequences in Milk from Water Buffaloes (Bubalus arnee f. bubalis)
Source: Viruses. 2021 Jun 7;13(6):1088. doi: 10.3390/v13061088 (PMC8228113; doi:10.3390/v13061088)
Supplement: Supplementary file 1 [file viruses-13-01088-s001.zip › viruses-1227574-supplementary/Figure S1.pdf]

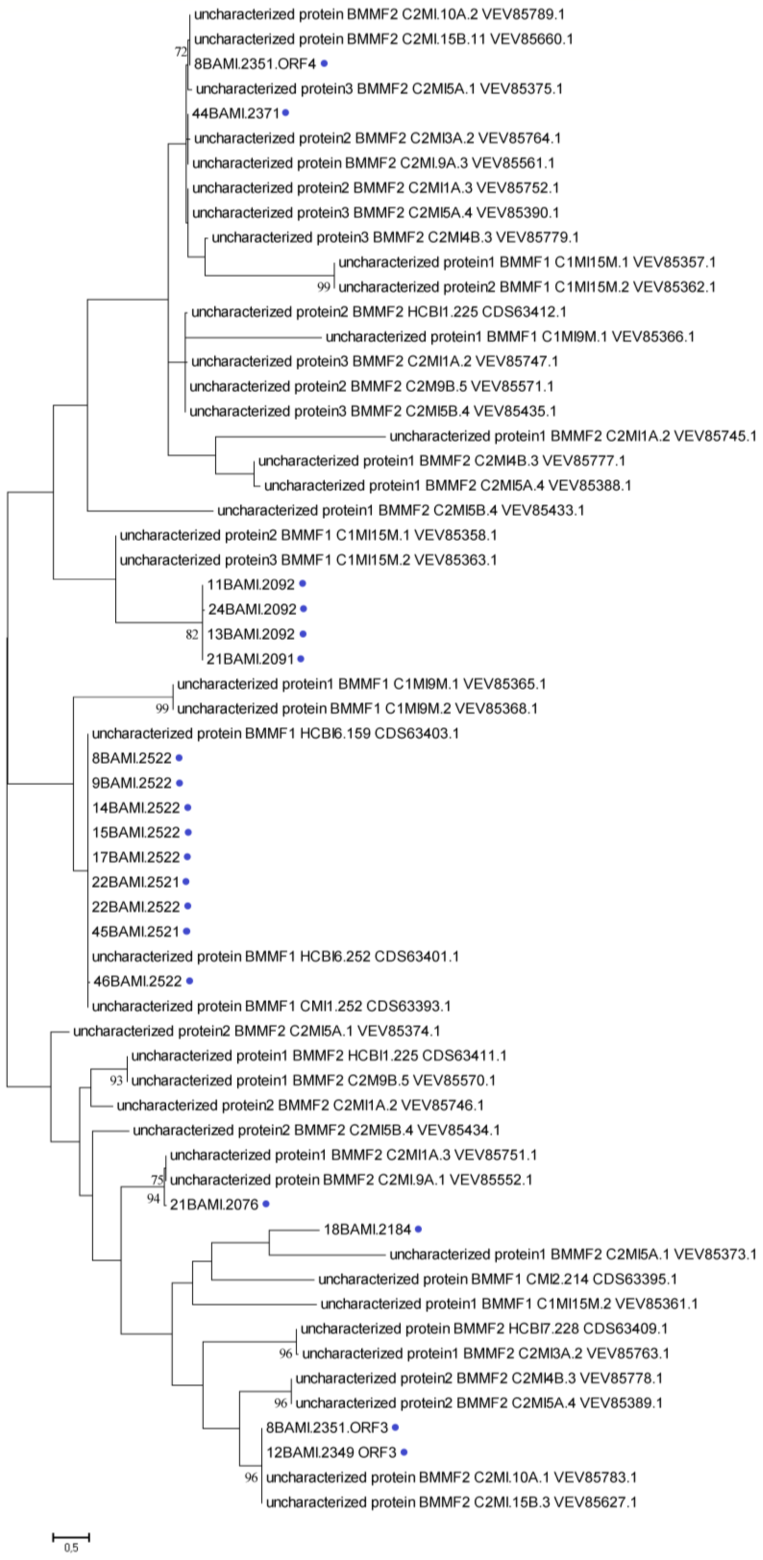

Figure S1. Maximum Likelihood phylogenetic tree of novel hypothetical protein sequences and uncharacterized BMMF1 and 2 proteins calculated as described in Fig. 1. The bootstrap consensus tree is based on 500 replicates. Bootstrap values at the branch points indicate the percentage of replicate trees in which respective isolates clustered. Branch support values lower than 70 % were not included. Blue dots mark all sequences found in this study. The scale bar represents the number of substitutions

per site. All taxa are indicated by name followed by the corresponding GenBank accession number.
